# Nutritional Components of Millet Porridge Cooked by Different Electric Cookers Based on Principal Component and Cluster Analyses

**DOI:** 10.3390/foods11182823

**Published:** 2022-09-13

**Authors:** Jiali Zhang, Pengliang Li, Junli Liu, Yunting Wang, Aixia Zhang, Wei Zhao, Shaohui Li, Yingying Liu, Jingke Liu

**Affiliations:** Institute of Biotechnology and Food Science, Hebei Academy of Agriculture and Forestry Sciences, 598 Heping West Rd., Xinhua, Shijiazhuang 050031, China

**Keywords:** millet porridge, electric cooker, nutritional composition, principal component analysis, cluster analysis

## Abstract

(1) Background: In order to study the effects of different electric cookers on the nutritional components of millet porridge, five different electric cookers (No. 1–5) were selected to cook millet porridge, then sensory and nutritional components in millet porridge, millet soup, and millet grains were analyzed; (2) Methods: Using principal component and cluster analysis, a variety of nutritional components were comprehensively compared; (3) Results: The results showed that among the different cooked samples, the content of amylose and reducing sugars was the highest in the samples cooked by electric cooker No. 3. The electric cooker No. 4 samples had the highest sensory evaluation score, crude fat, and protein content. The contents of ash, fatty acids, bound amino acids, and minerals were the highest in the electric cooker No. 5 samples. The sensory evaluation score and content of crude fat, ash, reducing sugars, direct starch, and Cu were higher in millet grains than in millet soup or porridge. The content of fatty acids, protein, amino acid, Zn, Fe, Mg, Mn, and Ca was highest in millet soup. Different electric cookers produced millet porridge with varying nutritional levels; (4) Conclusions: This study provides a reference for the further development of new electric cookers.

## 1. Introduction

Foxtail millet (*Setaria italica* (L.) *P. Beauv.*), is a gramineous dogtail crop originated from China and widely planted in northern China due to its short growth period and drought resistance [1,2]. It contains highly nutritional components [3,4,5], and plays a very important role in the Chinese diet. Among the consumption forms of millet, more than 80% is cooked into porridge [6,7].

Millet porridge contains a variety of nutrients, mainly including amino acids, protein [8], starch, and fatty acids [9]. It is a more complex system than rice porridge, including liquid and solid. In the process of cooking millet porridge, the starch is heated and gelatinized, and then cooled to produce a gelling system [10,11]. At present, the research on millet porridge mainly includes a focus on the functional components, volatile components and rheological quality [12]. For example, some scholars have found that millet porridge and the supernatant of millet porridge and granules of millet porridge can improve the gastrointestinal function of constipation model mice to a certain extent [13,14]. Some scholars have found that unsaturated fatty acids play important roles in the formation of the volatiles of foxtail millet porridge [15]. The rheological quality of pearl millet porridge was affected by the grain size. It was found that 0.841 mm pearl millet and 15% SMP were the most suitable for preparing qualified porridge [16]. However, there are few studies on the nutritional quality of millet porridge.

An electric cooker, which has been used to cooking rice, is designed to cook the millet porridge in. The porridge quality (aroma, nutrition, and texture) is affected by the temperature and pressure of the cooker [17,18]. Some scholars have studied that the rice quality is dependent on the forms of the rice, water-to-rice ratio, and the preset cooking method of the electric cooker [19]. Another study shows that the rice taste is influenced by the heating rate of the cooker [20]. That means the meals are greatly affected by the cookers, as different cookers provide different pressures, heating rates and temperature. Therefore, it is very important to study the quality of millet porridge of different electric cookers for the design of cookers. Due to the rapid development of electric cookers and the fact that millet porridge contains many nutritional components, the data can be processed with the help of principal component analysis (PCA) and cluster analysis(CA).

Because electric cookers are developed rapidly and there are many nutritional components in millet porridge, the data can be processed with the help of PCA and CA. PCA is an unsupervised technique that can calculate the principal components having the largest variance in order to reduce the dimensionality in data sets and allow the visualization of clusters [21]. CA is an unsupervised classification technology that was employed for characterizing similarities among samples [22]. Such methods have been widely used in the screening of quality indexes and comprehensive evaluations of fruits and vegetables, medicinal materials, and grains [23].

In this study, five different electric cookers were selected to cook millet porridge. The sensory evaluation, basic components, amino acids, fatty acids, and minerals of millet porridge, millet soup, and millet grains were measured and analyzed. The differences between nutritional components of millet porridge cooked by different electric cookers were analyzed by PCA and CA to provide a theoretical basis for the study of new electric cookers. This experiment is the first to study the different nutritional quality of millet porridge, millet soup and millet grains in different electric cookers, which has significance for the development of millet porridge and provides reference for the further development of new electric cookers.

## 2. Materials and Methods

### 2.1. Chemicals

Millet was purchased from the Jianjun agricultural and agricultural products processing plant (Shijiazhuang, China). High-temperature-resistant amylase was purchased from Novozyme (Denmark). Anhydrous glucose was purchased from Sigma (China). Methyl palmitate, methyl stearate, methyl oleate, methyl linoleate, and methyl linolenate were obtained from Sigma (China). Calcium, zinc, manganese, copper, iron, and magnesium standard stock solutions (10 mg/mL) were purchased from the national center of analysis and testing for nonferrous metals and electronic materials. Amino acids were purchased from Hitachi (Japan). Boron trifluoride-methanol solution (14% BF_3_-methanol) was purchased from CNW Technologies (Dusseldorf, Germany). N-hexane (chromatographic purity) was purchased from Fisher Chemical.

### 2.2. Preparation of Millet Porridge, Millet Soup, and Millet Grains

70 g of millet were weighed and washed twice with purified water. The millet was placed into five electric cookers (No. 1: multistage induction heating (IH) electromagnetic heating, rated power 900 W; No. 2: pressure IH electromagnetic heating, rated power 1100 W; No. 3: IH electromagnetic heating, rated power 820 W; No. 4: pressure IH electromagnetic heating, rated power 1300 W; and No. 5: pressure, chassis heating, Rated power 1100 W). The cookers were filled with purified water at a millet-to-water mass ratio of 1:14, the same ratio used in previous rice experiments [12], and boiled for 40 min to obtain millet porridge. Millet porridge was filtered with a sieve to obtain millet soup and millet grains. The millet porridge, millet soup, and millet grains were dried in a freeze-dryer (ALPHA1–2, Christ, Germany), and pressed through an 80 mesh sieve, then stored in a refrigerator at −20 °C until analysis.

### 2.3. Sensory Evaluation

Sensory evaluation was performed according to the methods of Yang et al. [24], with slight modifications. After cooking the millet porridge in the five electric cookers, 10–13 reviewers evaluated the sensory characteristics of millet porridge, millet soup, and millet grains, and scored each item according to the scoring standard. The comprehensive score was the sum of each index score (Table 1).

### 2.4. Determination of Basic Components

#### 2.4.1. Determination of Total Fat

Total fat in the samples was determined by Soxhlet extraction. Briefly, 1 g sample was filled in a filter paper bag and extracted by petroleum ether at 50 °C for 5 h. The lost weight of the sample was the total fat content.

#### 2.4.2. Determination of Ash

Ash was produced by incinerating dried samples at 850 °C for 1 h in a laboratory oven [25].

#### 2.4.3. Determination of Protein

Protein content in samples was determined by the Kjeldahl method [26]. A Tecator digestion system (Hilleroed, Denmark) and a K9860 automatic Kjeldahl nitrogen analyzer (Jinan Haineng Instrument Co., Ltd., Jinan, China), were used for the analysis. The sample size used in the Kjeldahl procedure was approximately 0.50 g. Samples were weighed and transferred into the Kjeldahl digestion flask containing 1.0 g of catalyst (prepared by mixing 0.6 g of K_2_SO_4_ and 0.4 g of CuSO_4_.·5H_2_O) and 10 mL of concentrated H_2_SO_4_. The samples were digested in a digestion furnace for about 4 h, and then analyzed for total Kjeldahl nitrogen. Total nitrogen was determined by titration with standardized HCL to a mixed indicator endpoint (1 mg/mL bromocresol green and 1 mg/mL methyl red, in ethanol volume concentration r = 950 mL/L).

#### 2.4.4. Determination of Amylose

Amylose content was determined using a previously published method by Zhu et al. [27] and Ramirez et al. [28], with modifications. A 100 mg defatted sample was dissolved in 10 mL potassium hydroxide solution (1 mol/L) and water bathed at 85 °C, the volume was adjusted to 50 mL with distilled water, and the sample was filtered through filter paper after standing for 20 min Next, 3 mL of filtrate was added to 25 mL of distilled water. The pH was adjusted to 3.0 with hydrochloric acid solution, and 0.5 mL of iodine reagent was added to the sample. Sample volumes were adjusted to 50 mL with distilled water. After standing at room temperature in the dark for 25 min, the absorbance of amylose was measured at 620 nm and 415 nm by UV spectrophotometer (SP-752, Shanghai, China), with distilled water as blank control. The amylose content of the sample was calculated according to the dual-wavelength standard curve of amylose.

#### 2.4.5. Determination of Reducing Sugars

The dinitrosalicylic acid (DNS) method with slight improvements based on the method of McCleary et al. [29] was used for the determination of reducing sugars. Two g of sample and 30 mL water were added to a 50 mL centrifuge tube, and thoroughly mixed with an oscillator for 1 min. The sample was incubated in a 50 °C water bath for 60 min, then centrifuged (Avanti j-301, Beckman Kool, Indianapolis, IN, USA) at 5000 rpm for 20 min and filtered. Next, 1 mL of supernatant was reacted with DNS, and the absorbance at 540 nm was measured with an ultraviolet spectrophotometer. The reducing sugars content of the sample was calculated according to the linear equation.

### 2.5. Determination of Fatty Acids

Fatty acids determination was based on a previously described method [30]. Briefly, 1 g of each sample was added to 30 mL of a solution of diethyl ether and petroleum ether (1:1, *v/v*) and thoroughly mixed in a 50 mL centrifuge tube for 2 h at 250 rpm using a shaker (MAXQ 4000, Thermo Scientific, Waltham, CA, USA). The mixture was then centrifuged at 4000 rpm for 10 min, and the supernatant was dried in a rotavapor at 35 °C (Buchi R215, Flawil, Switzerland). The dried residuals were redissolved in 1 mL of a 14% BF_3_-methanol solution and placed in a water bath at 80 °C for 2 min. The derivative reaction was terminated by placing the sample on ice, and the fatty acids methyl ester was extracted with 2 mL n-hexane.

The fatty acids methyl esters of palmitic acid, stearic acid, oleic acid, linoleic acid, and linolenic acid were determined by a method described in a previous study, with slight modifications, using a gas chromatography-flame ionization detector (GC-FID) (7820A, Agilent Technologies, Santa Clara, CA, USA) [30]. The injector temperature was 260 °C with splitless injection. An HP-INNOWAX column (30 m × 0.25 mm inner diameter and 0.25 μm film thickness, Agilent Technologies, Santa Clara, CA, USA) was used for analysis. The column initial temperature was 50 °C. The column was heated at 10 °C/min to 150 °C and held for 3 min The column was then heated at 4 °C/min to 205 °C and held for 1 min, then heated at 8 °C/min to the final temperature of 235 °C and held for 2 min The carrier gas (nitrogen, purity > 99.999%) was at a constant flow rate of 1 mL/min. The detector temperature was 260 °C. The airflow and hydrogen flow were 400 and 30 mL/min, respectively. A total of five external standard calibration curves were generated to quantify the fatty acids.

### 2.6. Determination of Amino Acids

Amino acid content was determined using a previously published method with a slight modification [31]. Two grams of each sample were added into a hydrolysis tube, and then 10 mL hydrochloric acid solution (6 mol/L) and four drops of phenol were mixed with the sample. Next, the sample was hydrolyzed for 22 h in a hydrolysis furnace, then removed and cooled to room temperature. The hydrolysate was filtered into a 50 mL volumetric flask, adjusted to a constant volume with distilled water, and shaken to mix evenly. One mL of the filtrate was then transferred into a 15-mL test tube and dried under reduced pressure with an evaporator. After drying, the residue was dissolved in 2 mL of distilled water, then dried under reduced pressure and evaporated to dry. The dried sample was dissolved with 2.0 mL of a sodium citrate buffer solution (pH 2.2), mixed well by shaking, and filtered through a 0.2-µm membrane. Finally, the sample was transferred into the instrument injection bottle for sample determination by the amino acid analyzer (L8900, Hitachi, Japan). The chromatographic column used sulfonic acid cationic resin, with detection wavelengths of 570 nm and 440 nm.

### 2.7. Determination Minerals

Minerals were determined according to a previously published method and slightly modified [32]. Five hundred mg of each sample were added into a conical flask, and then 10 mL H_2_NO_3_, 5 mL H_2_O_2_, and two drops of H_2_SO_4_ were mixed with the sample and incubated for 12 h. The flask funnel was covered and the sample evaporated to 1 mL using an adjustable electric heating plate. Next, 6 mL of nitric acid were added, and the sample was again evaporated to 1 mL and cooled, resulting in a colorless or yellowish solution. Next, 20 mL of ultrapure water was added. The sample was cooled, then diluted to 100 mL with ultrapure water. Flame atomic absorption spectrometry was used for mineral content determination (PinAAcle 900T, thermoelectricity, USA).

### 2.8. Data Processing Method

All experiments were repeated three times, and the data were expressed as mean ± standard error, which was calculated by dry mass. A statistical analysis was performed on the nutritional components of millet porridge and the electric cookers using the analysis of variance analysis (ANOVA) with Duncan’s multiple range tests. A *p*-value of 0.05 or less was considered to be statistically significant. The analyses were conducted using SPSS Statistics 25.0 (IBM, New York, NY, USA). SIMPCA-P software was used for PCA, and MultiExperience Viewer 4.9.0 software was used for CA.

## 3. Results and Discussion

### 3.1. Sensory Evaluation

Sensory evaluation of food is an intuitive index to describe and judge product quality [24]. Here, a sensory evaluation was performed on millet porridge, millet soup, and millet grains cooked by different electric cookers (Table 2). There were significant differences between millet porridge, millet soup, and millet grains cooked in the same electric cooker (*p* < 0.05), among which the uniformity, smell, texture, and total score of millet soup were higher. There were significant differences between millet soup, millet grains, and millet porridge cooked by different electric cookers (*p* < 0.05). Millet porridge sample 4 had a higher uniformity (19.81 ± 0.24) g/100 g, smell (21.52 ± 0.44) g/100 g, texture (21.07 ± 0.40) g/100 g, and total score (81.21 ± 1.39) g/100 g compared to the porridge prepared in the other cookers. The color and luster (20.36 ± 0.91) g/100 g, texture (22.27 ± 0.24) g/100 g, and total score (84.72 ± 1.06) g/100 g of millet soup sample 4 were higher than the other millet soup samples. The color and luster (21.10 ± 0.85) g/100 g, uniformity (20.53 ± 0.07) g/100 g, and total scores (80.55 ± 1.89) g/100 g of millet grain were higher in sample 4 than the other millet grain samples.

The probable reason for these results is that electric cooker No. 4 operated at the highest power and pressure of all the cookers. Under high temperature and high pressure, millet fully absorbed water and expanded, and lipids dissolved out of the grain, producing a millet porridge bright in color, sticky, and with a better taste. High pressure cooking can lead to a more uniform gel, and make the millet porridge mix more evenly [33,34]. At the same time, high temperature and high pressure also promoted the easier oxidation of free fatty acids to produce volatile odor substances, so that the aroma of millet porridge was rich. Peptides and free amino acids generated by proteolysis have been reported to bind volatile compounds such as butyral, octanal, and 2-pentanone, which is attributed to the reduction in molecular weight and the release of hydrophobic amino and carboxyl groups during proteolysis, thereby increasing their solubility and interaction with flavor compounds [35]. Under high pressure, millet grains absorb water and swell, making them taste better. Similar studies found that the sensory score of high-pressure cooked rice was also the highest compared to other cooking methods, which may be related to the high temperature and high pressure [19]. Some scholars have found that food cooked under high pressure had higher sensory scores [36], consistent with the results of this experimental study. In addition, some researchers found that the millet porridge cooked in a covered pan and a 1400 W induction cooker without pressure has the strongest foxtail millet porridge flavor, the best palatability, and the highest sensory score, which is basically similar to the results of this experiment [37].

### 3.2. Content Analysis of Basic Components

Protein, fat, and sugar are common nutrients in millet, and they are also indispensable substances for the human body. There were significant differences in these nutrients between millet porridge, millet soup, and millet grains cooked in the same electric cooker (*p* < 0.05) (Table 3). The content of ash, total fat, amylose, and reducing sugars in millet soup was higher than those in other portions, while the content of protein in millet grains was the highest. This indicates that most of the water-soluble nutrients in millet were transferred from the millet grains to the millet soup during the cooking process [38]. The denatured protein still existed in the millet grains due to its insolubility in water.

There were significant differences in millet porridge, millet soup, and millet grains cooked by different electric cookers (*p* < 0.05). The contents of ash, amylose, and reducing sugars were at the highest level in sample 3 (except the ash in millet grain 3 and amylose in millet porridge 3). The reason may be the low pressure and low power of electric cooker No. 3 [39]. The content of total fat and protein were at the highest level in sample 4 (except the total fat in millet soup). The reason might be due to the highest power, IH heating function and high pressure of the electric cooker No.4. The IH means that the cooker is heated around the entire surface using a tromagnetic heating coil, which results in the heating speed being faster and the food being heated more evenly. Moreover, higher pressure can provide a higher temperature of the porridge, and results in the release of the lipid and protein from the lipid-starch or protein-starch complex.

These substances are not only nutrients, but also carrier substances. Protein is the carrier of amino acids, and fat is the carrier of fatty acids and fat soluble vitamins. The content of amylose can affect the texture properties of starch gelatin [40]. At the same time, these carriers can also be combined with functional components to improve the scope of their application in functional foods. For example, polyphenols, polysaccharides and proteins form covalent or non-covalent compounds, and this combination will increase their physical stability, antioxidant activity and bioavailability [41]. The nutritional content of millet porridge cooked in different electric cookers is different, which lays the foundation for the future study of functional components.

### 3.3. Fatty Acids Content Analysis

The fatty acids in millet are mainly unsaturated fatty acids, accounting for 85.54% of the total fatty acids, which is similar to our previous research [42]. Unsaturated fatty acids have physiological functions of strengthening the brain, supporting intelligence, and delaying aging. Five fatty acids were detected in millet porridge: palmitic acid, stearic acid, oleic acid, linoleic acid, and linolenic acid (Table 4). The linoleic acid was the main fatty acid. The contents of the five fatty acids in millet grains, especially linoleic acid and oleic acid, were higher than those in millet porridge or soup. This shows that most fatty acids remained in millet grains during the cooking of millet porridge.

There were significant differences in millet porridge, millet soup, and millet grains cooked by different electric cookers (*p* < 0.05). The fatty acids were at the highest level in sample 5. The reason might be due to the lipid hydrolysis being enhanced with a high temperature and the high pressure of the electric cooker No. 5. Some scholars found that the content of fatty acids was apparently increased due to heat degradation during food heating [43], which is consistent with the above finding.

Moreover, the fatty acids are the main precursors of the aldehydes, ketones, and alkanes, and their degradation is a contribution to the formation of the aroma of millet porridge. Millet is rich in both free fatty acids and bound fatty acids. Bound fatty acids will decompose into free fatty acids during the heating process of millet porridge, promoting the increase of free fatty acids. Free fatty acids (especially unsaturated fatty acids) are unstable, which will further form hydroperoxide free radicals, and then trigger a series of cyclization, oxidation, cracking and other reactions, producing volatile components such as aldehydes, ketones, alcohols, and acids [15]. Zhang et al. [44] found that the volatile components of millet soup and millet porridge cooked in the same electric cooker are more similar, and the aroma is stronger, indicating that fatty acids are degraded into volatile components. The content of fatty acids in millet grain is higher, corresponding to the lighter aroma.

### 3.4. Amino Acid Content Analysis

Amino acids, the degradation products of proteins, can provide the rich nutritional quality and taste quality of millet porridge. The amino acid content of different millet products processed by five electric cookers was compared, and a total of 17 amino acids were found, seven of which were amino acids that are essential for human needs (Table 5).

The change trends of amino acids content in three products were similar, but the contents were different. The difference in amino acid content between millet porridge, millet soup and millet grains cooked in the same electric cooker was statistically significant (*p* < 0.05), and the order from large to small was millet grains > millet porridge > millet soup. The contents of total amino acids and essential amino acids were highest in millet grains, while the dilute and cooking treatment with water resulted in decreased amino acid concentrations in the supernatant and porridge. The content of essential amino acids in millet porridge and millet grains is high, and the ratio of essential amino acids (EAA) to total amino acids (TAA) is close to the value proposed by the Food and Agriculture Organization of the United Nations (FAO) (0.400), indicating that the proteins in millet porridge and millet grains are high-quality proteins with high nutritional value [45].

There were statistically significant differences among millet porridge, millet soup and millet grains cooked by different electric cookers (*p* < 0.05). The amino acid content of sample 4 and 5 was higher than that of the other three groups, which may because the temperature changed with the increase of pressure during cooking, and the hydrolysis rate of peptide bonds increased, which accelerated the thermal degradation of proteins, making the amino acid content in the system increase [46]. The comparison of sample 2 and 5 showed the high intensity of thermal degradation by chassis heating, although the study has demonstrated its thermal inhomogeneity. The comparison of sample 1 and 3 showed the advantage of multiterminal electromagnetic heating. Most amino acids of sample 4 were higher than sample 2, probably due to differences in thermal efficiency, proving the importance of processing parameters. Fu’s research has demonstrated that cooking can induce the formation of disulfide bonds and hydrophobic aggregates, as well as changes in protein secondary structure (transitions between *α*-helix, *β*-sheet, *β*-turn), giving millet a higher amino acid content and in vitro protein digestibility, thereby producing more bioactive amino acids or peptides during intestinal digestion, participating in the regulation of glucose metabolism and improving blood sugar levels [47]. However, some studies have also shown that the in vitro protein digestibility of millet is significantly reduced after high pressure steam treatment, mainly due to protein denaturation, reduced protein resistance to enzymes, and protein-other interactions [48]. Further research is necessary to confirm this.

### 3.5. Mineral Analysis

Minerals are essential to the human body. There were significant differences in Zn, Mn, Fe, and Mg in millet porridge, millet soup, and millet grains cooked in the same electric cooker (*p* < 0.05). The contents of minerals in millet grains was high (Table 6). It showed that the minerals were relatively stable and mainly located in the millet grains.

There were significant differences in the content of Zn, Mn, Fe, and Mg in millet porridge, millet soup, and millet grains cooked by different electric cookers (*p* < 0.05). The contents of the Zn, Mn, Fe, and Mg in sample 5 were the at highest level. However, Liang et al.’s research has shown that the contents of Ca and Mg in minerals of different varieties of millet were different from that of this study [49]. The possible reason is that different millet varieties cause different mineral content.

Feng reported that the content of Ca in 44 millet mineral elements in Inner Mongolia is 120.9~344.7 mg/kg, the content of Mg is 405.5~1306.7 mg/kg, the content of Fe is 7.69~31.07 mg/kg, the content range of Zn is 15.10~46.26 mg/kg, and the content range of Cu is 2.06~5.81 mg/kg [50]. The results are basically the same. The reason might be that the high temperature and high pressure of electric cooker No. 5 led to the breakage of the cell wall, resulting in the easy release of elemental minerals. Minerals exist in free form because of chelation combined with protein and starch. During cooking and heating, protein and starch can be separated out, which will have a certain effect on the quality of millet porridge.

### 3.6. Principal Component Analysis

**Figure 1:**
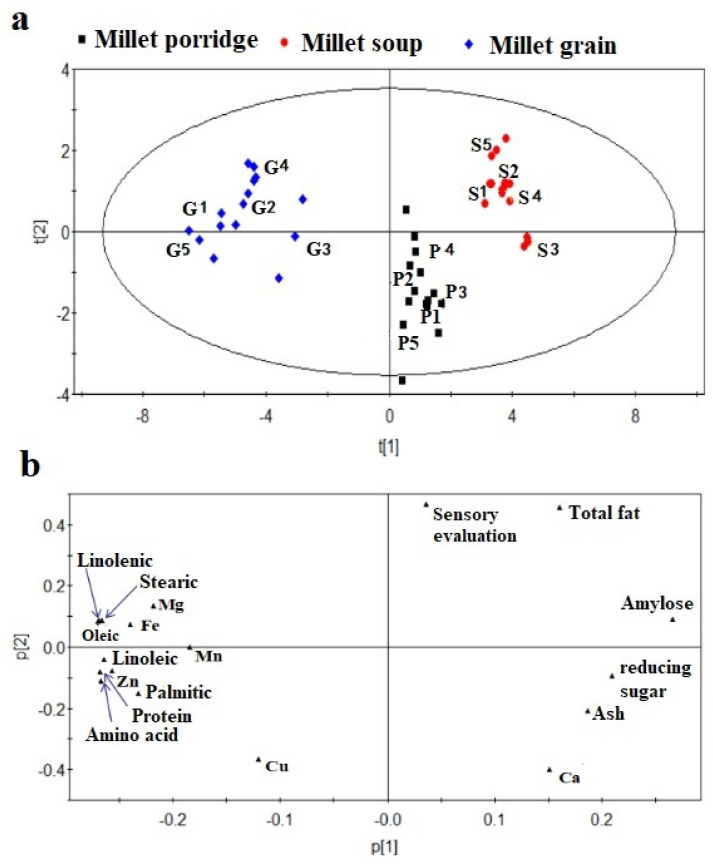
Principal component analysis (PCA) of millet porridge cooked in different electric cookers: (**a**) PCA score diagram; (**b**) PCA load diagram.

PCA was carried out on the nutritional components in the samples (Figure 1). The first principal component divided the samples into two groups: millet porridge and millet soup as a group, and millet grain as a group. The results showed that most of the nutrients of millet porridge and millet soup were similar. The second principal component divided the samples into two groups: millet grains and millet soup as a group, and millet porridge as a group. These results showed that some nutrients in millet grains and millet soup were similar (Figure 1a). The load diagram shows that the first principal component of millet soup mainly contained sensory evaluation, total fat, amylose, reducing sugars, ash, and Ca, and the samples of millet grain mainly contained protein, amino acids, fatty acids, Fe, Mn, Mg, Zn, and Cu (Figure 1b). This is consistent with the results of the significant difference analysis.

The main components of millet soup were amylose and reducing sugars. Amylose has a good ability to dissolve fat, and therefore the content of amylose and crude fat in millet soup was high. The millet grains absorbed water and expanded at high temperature, the lipids and minerals dissolved out of the grain, the hydrolysis of lipids was enhanced, and the content of fatty acids was increased. Therefore, fatty acids and minerals were mainly found in the millet grains.

### 3.7. Cluster Analysis

**Figure 2:**
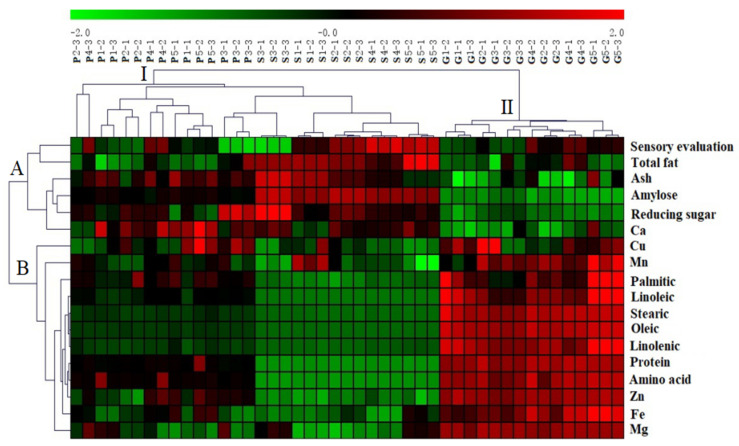
Cluster analysis of millet porridge cooked by different brands of electric cookers. The data are auto-scaled and clustered according to the Pearson correlation coefficients. Green colors indicate that the content level (or score for sensory evaluation) is less than its mean level, while red colors indicate that the content level (or score) is higher than its mean level.

CA of samples and compounds was carried out, as shown in Figure 2. Cluster analysis divided the samples into two groups. Group I was millet porridge and millet soup, indicating that the two were similar. Group II was millet grain, which showed that there was a great difference between millet grain compared to the millet porridge and soup. This is consistent with the PCA score map, with millet porridge and millet soup as one group and millet grain as another group (Figure 1a). The parameters evaluated for the three millet preparations were then divided into two groups by cluster analysis. Group A was higher in sensory evaluation, total fat, ash, reducing sugars, amylose and Ca, indicating that the content of these components or scores were higher in millet soup and lower in millet grain. Group B mainly contained palmitic acid, linoleic acid, stearic acid, oleic acid, linolenic acid, protein, amino acid, Zn, Fe, Mg, Mn, and Cu, indicating that the content of these components was higher in millet grain and the content of millet soup was the least, and the millet porridge content was intermediate between these. These results are consistent with the PCA map of principal components, which shows that the two statistical methods obtained the same analysis results (Figure 1b).

PCA is characterized by simplifying multiple variables into a few comprehensive variables, thus fully reflecting the overall information. CA is presented by heat map, which not only classifies the data, but also makes the results more intuitive through color comparison. The combination of principal component analysis and heat map analysis reveals the different nutritional components in millet porridge, millet soup and millet granules from different angles and dimensions. Most of the millet soup is the basic ingredient, and the millet grains contain protein, fatty acids, amino acids and minerals, while the nutritional components of the millet porridge is between the the millet soup and the millet grains. Therefore, it provides a certain reference for the future study of millet porridge functional components.

## 4. Conclusions

In this study, five electric cookers were selected. Under the same cooking conditions, PCA and cluster analysis were used to examine the differences between various nutrients in millet porridge, millet soup, and millet grains cooked by different electric cookers. The results showed that among the samples cooked in different electric cookers, the samples cooked in electric cooker No. 3 had the highest content of amylose and reducing sugars. The sensory evaluation, total fat, and protein content of the samples cooked in electric cooker No. 4 were the highest. The samples cooked in electric cooker No. 5 had the highest content of ash, fatty acids, amino acids, and minerals. The sensory evaluation score and content of total fat, ash, reducing sugars, amylose, and Cu in millet soup were higher than that of millet porridge or grain. The content of fatty acids, protein, amino acids, Zn, Fe, Mg, Mn, and Ca in millet grain was higher than that of porridge or soup. The content of millet porridge was generally between millet soup and grain. The nutritional components of millet grain, millet soup, and millet porridge cooked in different electric cookers varied, which may be related to the different heating modes and power of the electric cookers. This study provides a reference for the further development of new electric cookers.

## Figures and Tables

**Table 1:** Scoring rules for sensory evaluation of millet porridge.

Index	Percent	Standard Score
Color and luster	25	The color is golden yellow and slightly shiny (21–25 points) The color is light yellow with a slight luster (16–20 points) The color is dark and dull (10–15 points)
Uniformity	25	The millet porridge is not layered, the millet soup is evenly distributed, the millet grains are moderately expanded, and there is almost no damage, or the damage rate is very low (21–25 points) The millet porridge is slightly layered, the millet soup is turbid, the millet grains have a certain degree of expansion and a certain damage rate (16–20 points) The millet porridge has obvious stratifications, the millet soup is clear, the water absorption and expansion of the millet grains are low, and the damage rate is high (10–15 points)
Smell	25	There is a unique and strong aroma (21–25 points) There is a light fragrance with no peculiar odor (16–20 points) There is no fragrance but there is a peculiar odor (10–15 points)
Texture	25	The taste is pleasing with a smooth texture (21–25 points) The taste is average with a slightly rough texture (16–20 points) There is a rough texture (10–15 points)
Total score	100	

**Table 2:** Sensory evaluation results of samples cooked in different electric cookers.

Number	Color and Luster	Uniformity	Smell	Texture	Total Score
Millet porridge 1	19.53 ± 0.39 ^bA^	17.20 ± 0.18 ^dC^	19.33 ± 0.21 ^bB^	19.35 ± 0.18 ^cB^	75.41 ± 1.22 ^cB^
Millet porridge 2	19.90 ± 0.76 ^aB^	17.82 ± 0.29 ^cC^	17.66 ± 0.22 ^cC^	18.83 ± 0.57 ^dB^	74.21 ± 1.02 ^dC^
Millet porridge 3	18.61 ± 0.44 ^dC^	16.23 ± 0.30 ^eC^	17.05 ± 0.32 ^dC^	18.90 ± 0.32 ^dA^	70.78 ± 1.59 ^eB^
Millet porridge 4	18.82 ± 0.88 ^cC^	19.81 ± 0.24 ^aC^	21.52 ± 0.44 ^aA^	21.07 ± 0.40 ^aB^	81.21 ± 1.39 ^aB^
Millet porridge 5	18.83 ± 0.85 ^cC^	18.63 ± 0.13 ^bC^	19.62 ± 0.29 ^bB^	19.72 ± 0.35 ^bB^	76.79 ± 1.33 ^bC^
Millet soup 1	19.27 ± 0.74 ^cB^	19.73 ± 0.20 ^bA^	19.27 ± 0.87 ^cB^	20.74 ± 0.26 ^bA^	79.02 ± 1.40 ^dA^
Millet soup 2	18.09 ± 0.72 ^dC^	21.55 ± 0.46 ^aA^	20.08 ± 0.14 ^bA^	22.26 ± 0.24 ^aA^	81.98 ± 1.32 ^cA^
Millet soup 3	16.82 ± 0.23 ^eB^	17.09 ± 0.77 ^cA^	19.74 ± 0.90 ^bA^	17.02 ± 0.65 ^cC^	70.68 ± 1.30 ^eB^
Millet soup 4	20.36 ± 0.91 ^aB^	21.51 ± 0.46 ^aA^	20.55 ± 0.34 ^aB^	22.27 ± 0.24 ^aA^	84.72 ± 1.06 ^aA^
Millet soup 5	19.55 ± 0.69 ^bA^	21.64 ± 0.29 ^aA^	20.73 ± 0.37 ^aA^	21.99 ± 0.08 ^aA^	83.92 ± 1.50 ^bA^
Millet grain 1	18.62 ± 0.28 ^eC^	18.82 ± 0.71 ^eB^	20.12 ± 0.46 ^aA^	17.42 ± 0.28 ^cC^	74.99 ± 1.08 ^dB^
Millet grain 2	20.92 ± 0.47 ^bA^	19.12 ± 0.31 ^dB^	19.42 ± 0.43 ^bB^	18.22 ± 0.17 ^bC^	77.68 ± 1.80 ^cB^
Millet grain 3	18.67 ± 0.85 ^dC^	19.78 ± 0.10 ^cB^	18.37 ± 0.93 ^cB^	18.08 ± 0.32 ^bB^	74.90 ± 1.36 ^dA^
Millet grain 4	21.10 ± 0.85 ^aA^	20.53 ± 0.07 ^aB^	19.70 ± 0.70 ^bC^	19.22 ± 0.53 ^aC^	80.55 ± 1.89 ^aB^
Millet grain 5	19.40 ± 0.17 ^cB^	20.20 ± 0.69 ^bB^	19.51 ± 0.57 ^bB^	19.30 ± 0.20 ^aC^	78.42 ± 0.88 ^bB^

Note: Data were shown as the mean ± SD values (*n* = 3, g/100 g dry basis). Different lowercase letters in the same column indicate that there were significant differences when cooking the same sample in different electric cookers (*p* < 0.05). Different capital letters in the same column indicate that the samples cooked in the same electric cooker had significant differences (*p* < 0.05).

**Table 3:** Content of basic components of millet preparations cooked in different electric cookers.

Sample Name	Ash	Total Fat	Amylose	Protein	Reducing Sugars
Millet porridge 1	0.75 ± 0.03 ^bA^	3.48 ± 0.06 ^bB^	17.47 ± 0.18 ^abB^	8.63 ± 0.19 ^bcB^	0.36 ± 0.04 ^cA^
Millet porridge 2	0.77 ± 0.06 ^abB^	3.70 ± 0.16 ^bC^	16.47 ± 0.18 ^bcB^	8.82 ± 0.01 ^bB^	0.44 ± 0.03 ^bB^
Millet porridge 3	0.82 ± 0.03 ^aB^	3.71 ± 0.11 ^aAB^	16.18 ± 0.03 ^cB^	8.55 ± 0.12 ^cB^	0.64 ± 0.03 ^aB^
Millet porridge 4	0.73 ± 0.07 ^bA^	4.33 ± 0.10 ^aA^	16.50 ± 0.19 ^bcB^	9.27 ± 0.03 ^aB^	0.43 ± 0.01 ^bA^
Millet porridge 5	0.78 ± 0.06 ^abA^	3.67 ± 0.07 ^bB^	17.88 ± 0.19 ^aB^	9.14 ± 0.06 ^aB^	0.26 ± 0.04 ^dB^
Millet soup 1	0.92 ± 0.02 ^bA^	5.46 ± 0.03 ^bA^	24.41 ± 0.16 ^cA^	2.94 ± 0.06 ^aC^	0.41 ± 0.04 ^cA^
Millet soup 2	0.85 ± 0.05 ^cA^	5.28 ± 0.28 ^cA^	26.75 ± 0.17 ^bA^	2.85 ± 0.01 ^bC^	0.51 ± 0.02 ^bA^
Millet soup 3	0.97 ± 0.01 ^aA^	5.41 ± 0.33 ^bcA^	27.64 ± 0.20 ^aA^	2.80 ± 0.01 ^bC^	0.78 ± 0.01 ^aA^
Millet soup 4	0.81 ± 0.06 ^dA^	4.84 ± 0.16 ^dA^	24.77 ± 0.10 ^cA^	3.00 ± 0.04 ^aC^	0.43 ± 0.02 ^cA^
Millet soup 5	0.75 ± 0.02 ^eA^	6.12 ± 0.16 ^aA^	24.31 ± 0.13 ^cA^	2.86 ± 0.05 ^bC^	0.42 ± 0.03 ^cA^
Millet grain 1	0.62 ± 0.04 ^bB^	3.81 ± 0.15 ^bB^	9.20 ± 0.14 ^bC^	15.03 ± 0.22 ^bA^	0.21 ± 0.04 ^cB^
Millet grain 2	0.63 ± 0.02a ^bC^	4.20 ± 0.05 ^abB^	8.82 ± 0.10 ^cC^	16.28 ± 0.21 ^aA^	0.25 ± 0.01 ^bC^
Millet grain 3	0.74 ± 0.02 ^abC^	3.85 ± 0.18 ^bB^	10.84 ± 0.22 ^aC^	14.61 ± 0.22 ^bA^	0.30 ± 0.01 ^aC^
Millet grain 4	0.70 ± 0.05 ^abA^	4.77 ± 0.10 ^aA^	7.27 ± 0.22 ^dC^	16.71 ± 0.13 ^aA^	0.23 ± 0.01 ^bcB^
Millet grain 5	0.79 ± 0.02 ^aA^	3.72 ± 0.21 ^bB^	7.26 ± 0.19 ^dC^	16.16 ± 0.09 ^aA^	0.22 ± 0.02 ^cB^

Note: Data were shown as the mean ± SD values (*n* = 3, g/100 g dry basis). Significant differences between table entries are explained in the footnote of Table 2.

**Table 4:** Content of fatty acids in millet porridge cooked in different electric cookers.

Sample Name	Palmitic	Stearic	Oleic	Linoleic	Linolenic
Millet porridge 1	5.09 ± 0.03 ^bB^	4.71 ± 0.02 ^aB^	10.72 ± 0.02 ^cB^	22.31 ± 0.17 ^cB^	1.47 ± 0.06 ^bB^
Millet porridge 2	4.36 ± 0.03 ^dB^	4.37 ± 0.03 ^bB^	10.03 ± 0.03 ^bB^	19.26 ± 0.20 ^bB^	1.17 ± 0.02 ^abB^
Millet porridge 3	3.91 ± 0.05 ^eA^	4.02 ± 0.02 ^cB^	9.38 ± 0.03 ^bcB^	13.37 ± 0.16 ^aB^	1.06 ± 0.04 ^abB^
Millet porridge 4	4.58 ± 0.04 ^cA^	4.47 ± 0.03 ^bB^	10.58 ± 0.02 ^bcB^	13.86 ± 0.22 ^aB^	1.05 ± 0.01 ^abB^
Millet porridge 5	6.53 ± 0.06 ^aB^	4.73 ± 0.03 ^aB^	11.23 ± 0.04 ^aB^	26.08 ± 0.04 ^aB^	1.56 ± 0.05 ^aB^
Millet soup 1	3.16 ± 0.04 ^bC^	1.39 ± 0.01 ^aC^	1.64 ± 0.02 ^aC^	5.18 ± 0.04 ^bC^	0.29 ± 0.01 ^cB^
Millet soup 2	3.05 ± 0.05 ^bC^	1.34 ± 0.01 ^aC^	1.62 ± 0.01 ^aC^	5.21 ± 0.03 ^bC^	0.31 ± 0.05 ^cC^
Millet soup 3	3.43 ± 0.03 ^aC^	1.50 ± 0.01 ^aC^	1.69 ± 0.02 ^aC^	5.44 ± 0.06 ^aC^	0.32 ± 0.04 ^cC^
Millet soup 4	3.50 ± 0.02 ^aB^	1.46 ± 0.02 ^aC^	1.64 ± 0.03 ^aC^	5.42 ± 0.02 ^aC^	0.37 ± 0.03 ^bC^
Millet soup 5	3.57 ± 0.02 ^aC^	1.37 ± 0.03 ^aC^	1.71 ± 0.02 ^aC^	5.24 ± 0.01 ^bC^	0.42 ± 0.05 ^aC^
Millet grain 1	6.88 ± 0.02 ^bA^	7.96 ± 0.02 ^aA^	19.77 ± 0.02 ^bA^	39.48 ± 0.09 ^bA^	2.21 ± 0.05 ^bA^
Millet grain 2	5.69 ± 0.03 ^bcA^	7.44 ± 0.03 ^abA^	18.47 ± 0.03 ^bcA^	31.10 ± 0.03 ^cA^	1.96 ± 0.07 ^cA^
Millet grain 3	4.51 ± 0.02 ^cB^	6.67 ± 0.01 ^bA^	17.21 ± 0.02 ^cA^	22.02 ± 0.01 ^dA^	1.71 ± 0.08 ^dA^
Millet grain 4	5.69 ± 0.02 ^bcA^	7.88 ± 0.03 ^aA^	19.70 ± 0.02 ^abA^	28.83 ± 0.06 ^cA^	1.69 ± 0.03 ^dA^
Millet grain 5	9.05 ± 0.04 ^aA^	8.10 ± 0.05 ^aA^	21.06 ± 0.10 ^aA^	46.79 ± 0.09 ^aA^	2.60 ± 0.01 ^aA^

Note: Data were shown as the mean ± SD values (*n* = 3, mg/100 g dry basis). Significant differences between table entries are explained in the footnote of Table 2.

**Table 5:** Amino acid content of millet porridge cooked in different electric cookers.

**Sample Name**	**Aspartic Acid**	**Serine**	**Glutamate**	**Glycine**	**Alanine**	**Cystine**	**Tyrosine**	**Proline**	**Arginine**	**Threonine**
Millet porridge 1	0.62 ± 0.01 ^aB^	0.46 ± 0.01 ^aB^	2.00 ± 0.03 ^aB^	0.23 ± 0.01 ^aB^	0.23 ± 0.05 ^bB^	0.12 ± 0.01 ^abA^	0.54 ± 0.02 ^aA^	0.66 ± 0.01 ^aB^	0.17 ± 0.01 ^bB^	0.35 ± 0.01 ^aB^
Millet porridge 2	0.62 ± 0.01 ^aB^	0.46 ± 0.01 ^aB^	2.00 ± 0.06 ^aB^	0.23 ± 0.01 ^aB^	0.25 ± 0.11 ^bB^	0.16 ± 0.03 ^aA^	0.55 ± 0.05 ^aA^	0.67 ± 0.01 ^aB^	0.16 ± 0.01 ^bB^	0.36 ± 0.01 ^aB^
Millet porridge 3	0.60 ± 0.02 ^aB^	0.45 ± 0.01 ^aB^	1.96 ± 0.06 ^aB^	0.22 ± 0.01 ^bB^	0.32 ± 0.01 ^abB^	0.11 ± 0.01 ^bA^	0.51 ± 0.02 ^aA^	0.66 ± 0.01 ^aB^	0.16 ± 0.01 ^bB^	0.35 ± 0.01 ^aB^
Millet porridge 4	0.62 ± 0.01 ^aB^	0.45 ± 0.01 ^aB^	1.98 ± 0.01 ^aB^	0.23 ± 0.01 ^aB^	0.27 ± 0.11 ^bB^	0.14 ± 0.02 ^abA^	0.52 ± 0.03 ^aB^	0.66 ± 0.01 ^aB^	0.16 ± 0.01 ^bB^	0.35 ± 0.01 ^aB^
Millet porridge 5	0.62 ± 0.02 ^aB^	0.46 ± 0.02 ^aB^	2.04 ± 0.07 ^aB^	0.23 ± 0.01 ^aB^	0.41 ± 0.04 ^aB^	0.11 ± 0.04 ^bA^	0.53 ± 0.06 ^aB^	0.67 ± 0.03 ^aB^	0.20 ± 0.04 ^aB^	0.36 ± 0.01 ^aB^
Millet soup 1	0.20 ± 0.01 ^bC^	0.13 ± 0.01 ^abC^	0.53 ± 0.02 ^abC^	0.10 ± 0.01 ^aC^	0.24 ± 0.01 ^aB^	0.01 ± 0.01 ^cC^	0.09 ± 0.02 ^cC^	0.17 ± 0.01 ^aC^	0.06 ± 0.01 ^bC^	0.11 ± 0.01 ^aC^
Millet soup 2	0.20 ± 0.01 ^bC^	0.12 ± 0.01 ^bcC^	0.50 ± 0.03 ^bcC^	0.09 ± 0.01 ^bC^	0.21 ± 0.02 ^abB^	0.01 ± 0.01 ^cC^	0.08 ± 0.02 ^cB^	0.16 ± 0.02 ^aC^	0.06 ± 0.01 ^bC^	0.10 ± 0.01 ^bC^
Millet soup 3	0.19 ± 0.01 ^bC^	0.11 ± 0.01 ^cC^	0.47 ± 0.01 ^cC^	0.09 ± 0.01 ^bC^	0.18 ± 0.02 ^bcC^	0.01 ± 0.01 ^cB^	0.09 ± 0.01 ^cB^	0.13 ± 0.01 ^bC^	0.06 ± 0.01 ^bC^	0.09 ± 0.01 ^bC^
Millet soup 4	0.21 ± 0.01 ^abC^	0.13 ± 0.01 ^abC^	0.54 ± 0.01 ^aC^	0.10 ± 0.01 ^aC^	0.14 ± 0.04 ^cB^	0.03 ± 0.01 ^bB^	0.14 ± 0.02 ^bC^	0.15 ± 0.01 ^aC^	0.06 ± 0.01 ^bC^	0.11 ± 0.01 ^aC^
Millet soup 5	0.22 ± 0.01 ^aC^	0.14 ± 0.01 ^aC^	0.54 ± 0.02 ^aC^	0.10 ± 0.01 ^aC^	0.14 ± 0.04 ^cC^	0.04 ± 0.01 ^aB^	0.20 ± 0.03 ^aC^	0.15 ± 0.02 ^aC^	0.07 ± 0.01 ^aC^	0.11 ± 0.01 ^aC^
Millet grain 1	0.96 ± 0.01 ^bcA^	0.75 ± 0.01 ^bcA^	3.30 ± 0.03 ^bcA^	0.34 ± 0.01 ^abA^	1.56 ± 0.01 ^aA^	0.04 ± 0.01 ^cB^	0.40 ± 0.03 ^cB^	1.15 ± 0.01 ^bcA^	0.27 ± 0.01 ^abA^	0.57 ± 0.01 ^bcA^
Millet grain 2	0.94 ± 0.12 ^cA^	0.74 ± 0.09 ^cA^	3.21 ± 0.01 ^cA^	0.33 ± 0.04 ^bA^	1.42 ± 0.19 ^abA^	0.07 ± 0.03 ^bcB^	0.54 ± 0.09 ^bA^	1.09 ± 0.05 ^cA^	0.27 ± 0.03 ^abA^	0.56 ± 0.07 ^cA^
Millet grain 3	0.87 ± 0.04 ^cA^	0.68 ± 0.03 ^cA^	2.95 ± 0.03 ^cA^	0.31 ± 0.02 ^bA^	1.28 ± 0.02 ^bA^	0.09 ± 0.02 ^abA^	0.54 ± 0.09 ^bA^	1.01 ± 0.03 ^cA^	0.24 ± 0.02 ^bA^	0.52 ± 0.02 ^cA^
Millet grain 4	1.07 ± 0.06 ^abA^	0.84 ± 0.05 ^abA^	3.68 ± 0.20 ^abA^	0.38 ± 0.02 ^aA^	1.59 ± 0.14 ^aA^	0.11 ± 0.01 ^aA^	0.71 ± 0.03 ^aA^	1.26 ± 0.08 ^abA^	0.30 ± 0.01 ^aA^	0.64 ± 0.03 ^abA^
Millet grain 5	1.09 ± 0.01 ^aA^	0.85 ± 0.01 ^aA^	3.75 ± 0.07 ^aA^	0.38 ± 0.01 ^aA^	1.62 ± 0.02 ^aA^	0.11 ± 0.01 ^aA^	0.70 ± 0.02 ^aA^	1.30 ± 0.02 ^aA^	0.30 ± 0.01 ^aA^	0.65 ± 0.01 ^aA^
**Sample Name**	**Valine**	**Methionine**	**Isoleucine**	**Leucine**	**Phenylalanine**	**Lysine**	**Histidine**	**TAA**	**EAA**	**EAA/TAA**
Millet porridge 1	0.47 ± 0.01 ^abB^	0.20 ± 0.06 ^aB^	0.37 ± 0.01 ^aB^	1.32 ± 0.02 ^aB^	0.80 ± 0.10 ^aB^	0.14 ± 0.01 ^aB^	0.13 ± 0.01 ^aB^	8.81 ± 0.09 ^aB^	3.65 ^aB^	0.42 ^aA^
Millet porridge 2	0.47 ± 0.01 ^abB^	0.18 ± 0.01 ^aB^	0.37 ± 0.01 ^aB^	1.32 ± 0.04 ^aB^	0.75 ± 0.09 ^aB^	0.14 ± 0.01 ^aB^	0.13 ± 0.01 ^aB^	8.83 ± 0.19 ^aB^	3.59 ^aB^	0.41 ^aA^
Millet porridge 3	0.46 ± 0.01 ^bB^	0.17 ± 0.05 ^aB^	0.36 ± 0.01 ^aB^	1.30 ± 0.04 ^aB^	0.71 ± 0.11 ^aB^	0.14 ± 0.01 ^aB^	0.12 ± 0.02 ^aB^	8.59 ± 0.25 ^aB^	3.48 ^aB^	0.41 ^aA^
Millet porridge 4	0.47 ± 0.01 ^abB^	0.21 ± 0.02 ^aB^	0.37 ± 0.01 ^aB^	1.30 ± 0.01 ^aB^	0.74 ± 0.10 ^aB^	0.14 ± 0.01 ^aB^	0.12 ± 0.01 ^aB^	8.73 ± 0.08 ^aB^	3.59 ^aB^	0.41 ^aA^
Millet porridge 5	0.48 ± 0.01 ^aB^	0.19 ± 0.05 ^aB^	0.38 ± 0.01 ^aB^	1.34 ± 0.04 ^aB^	0.67 ± 0.03 ^aB^	0.15 ± 0.01 ^aB^	0.13 ± 0.01 ^aB^	8.97 ± 0.29 ^aB^	3.57 ^aB^	0.40 ^aA^
Millet soup 1	0.14 ± 0.01 ^aC^	0.01 ± 0.01 ^bcC^	0.11 ± 0.01 ^aC^	0.32 ± 0.01 ^aC^	0.07 ± 0.01 ^aC^	0.05 ± 0.01 ^aC^	0.07 ± 0.01 ^aC^	2.41 ± 0.06 ^abC^	0.82 ^abC^	0.34 ^bC^
Millet soup 2	0.13 ± 0.01 ^bC^	0.01 ± 0.01 ^cC^	0.10 ± 0.0 ^aC^	0.30 ± 0.02 ^bC^	0.06 ± 0.02 ^aC^	0.05 ± 0.03 ^aC^	0.06 ± 0.01 ^aC^	2.25 ± 0.17 ^bcC^	0.75 ^bcC^	0.33 ^bB^
Millet soup 3	0.12 ± 0.01 ^cC^	0.01 ± 0.01 ^bcC^	0.10 ± 0.01 ^aC^	0.29 ± 0.01 ^bC^	0.05 ± 0.01 ^aC^	0.05 ± 0.02 ^aC^	0.06 ± 0.01 ^aC^	2.11 ± 0.05 ^cC^	0.71 ^cC^	0.33 ^bC^
Millet soup 4	0.14 ± 0.01 ^aC^	0.03 ± 0.02 ^abC^	0.11 ± 0.02 ^aC^	0.34 ± 0.01 ^aC^	0.07 ± 0.01 ^aC^	0.05 ± 0.01 ^aC^	0.06 ± 0.01 ^aC^	2.42 ± 0.06 ^abC^	0.86 ^aC^	0.36 ^aC^
Millet soup 5	0.14 ± 0.01 ^aC^	0.04 ± 0.01 ^aC^	0.11 ± 0.01 ^aC^	0.33 ± 0.02 ^aC^	0.07 ± 0.04 ^aC^	0.05 ± 0.01 ^aC^	0.06 ± 0.01 ^aC^	2.53 ± 0.11 ^aC^	0.85 ^aC^	0.34 ^bB^
Millet grain 1	0.75 ± 0.01 ^bA^	0.40 ± 0.02 ^aA^	0.61 ± 0.01 ^bA^	2.21 ± 0.02 ^bcA^	1.10 ± 0.04 ^bA^	0.21 ± 0.01 ^abA^	0.25 ± 0.01 ^aA^	14.87 ± 0.17 ^bcA^	5.85 ^aA^	0.39 ^aB^
Millet grain 2	0.73 ± 0.09 ^bA^	0.35 ± 0.13 ^abA^	0.59 ± 0.08 ^bA^	2.15 ± 0.27 ^cA^	1.17 ± 0.13 ^abA^	0.19 ± 0.02 ^bcA^	0.25 ± 0.03 ^aA^	14.60 ± 0.23 ^cA^	5.74 ^aA^	0.39 ^aA^
Millet grain 3	0.68 ± 0.03 ^bA^	0.33 ± 0.04 ^bA^	0.55 ± 0.03 ^bA^	1.98 ± 0.09 ^cA^	1.08 ± 0.10 ^bA^	0.18 ± 0.07 ^cA^	0.22 ± 0.01 ^bA^	13.50 ± 0.34 ^cA^	5.31 ^aA^	0.39 ^aB^
Millet grain 4	0.85 ± 0.04 ^aA^	0.37 ± 0.06 ^aA^	0.68 ± 0.04 ^aA^	2.46 ± 0.13 ^abA^	1.23 ± 0.06 ^abA^	0.21 ± 0.01 ^abA^	0.26 ± 0.02 ^aA^	16.62 ± 0.78 ^abA^	6.44 ^aA^	0.39 ^aB^
Millet grain 5	0.85 ± 0.01 ^aA^	0.31 ± 0.01 ^bA^	0.69 ± 0.01 ^aA^	2.51 ± 0.04 ^aA^	1.27 ± 0.08 ^aA^	0.22 ± 0.01 ^aA^	0.25 ± 0.01 ^aA^	16.87 ± 0.23 ^aA^	6.51 ^aA^	0.39 ^aA^

Note: Data were shown as the mean ± SD values (*n* = 3 g/100 g dry basis). Significant differences between table entries are explained in the footnote of Table 2. TAA: Total Amino Acids. EAA: Essential Amino Acids.

**Table 6:** Mineral content in millet porridge cooked in different electric cookers.

Sample Name	Ca	Zn	Mn	Cu	Fe	Mg
Millet porridge 1	468.53 ± 0.39 ^aA^	27.53 ± 0.02 ^bB^	13.33 ± 0.00 ^aB^	2.13 ± 0.00 ^aA^	54.47 ± 0.01 ^cB^	554.48 ± 0.32 ^aB^
Millet porridge 2	404.67 ± 0.38 ^aA^	24.67 ± 0.06 ^cB^	13.32 ± 0.00 ^aB^	1.87 ± 0.00 ^aA^	64.07 ± 0.06 ^bB^	515.08 ± 0.40 ^aB^
Millet porridge 3	408.13 ± 0.27 ^aA^	31.27 ± 0.00 ^aB^	13.60 ± 0.00 ^aB^	2.60 ± 0.00 ^aA^	53.87 ± 0.03 ^cB^	521.09 ± 0.50 ^aC^
Millet porridge 4	418.33 ± 0.50 ^aA^	32.67 ± 0.01 ^aB^	14.07 ± 0.00 ^aB^	1.67 ± 0.00 ^aAB^	68.33 ± 0.07 ^bB^	546.28 ± 0.47 ^aB^
Millet porridge 5	554.13 ± 0.56 ^aA^	33.13 ± 0.01 ^aB^	14.20 ± 0.00 ^aB^	2.87 ± 0.01 ^aA^	77.07 ± 0.02 ^aB^	644.43 ± 0.14 ^aA^
Millet soup 1	325.87 ± 0.08 ^bA^	22.20 ± 0.00 ^bC^	16.07 ± 0.01 ^aA^	2.07 ± 0.00 ^aA^	52.60 ± 0.05 ^cB^	424.32 ± 0.04 ^cC^
Millet soup 2	416.00 ± 0.16 ^aA^	24.40 ± 0.00 ^abB^	13.27 ± 0.00 ^bB^	1.80 ± 0.00 ^abA^	60.13 ± 0.02 ^bB^	428.74 ± 0.12 ^bcC^
Millet soup 3	436.20 ± 0.09 ^aA^	23.53 ± 0.00 ^abB^	11.93 ± 0.00 ^cdC^	1.40 ± 0.00 ^abA^	52.47 ± 0.01 ^cB^	583.06 ± 0.10 ^aAC^
Millet soup 4	400.20 ± 0.16 ^aA^	24.93 ± 0.01 ^aC^	13.07 ± 0.00 ^bcC^	1.47 ± 0.00 ^abB^	46.40 ± 0.04 ^cC^	458.47 ± 0.22 ^bB^
Millet soup 5	429.00 ± 0.19 ^aA^	23.07 ± 0.01 ^abC^	11.00 ± 0.01 ^dC^	1.27 ± 0.00 ^bA^	68.20 ± 0.03 ^aB^	587.92 ± 0.21 ^aB^
Millet grain 1	252.00 ± 0.12 ^bB^	38.20 ± 0.01 ^abA^	13.73 ± 0.00 ^dB^	2.60 ± 0.00 ^aA^	78.53 ± 0.01 ^cA^	680.98 ± 0.11 ^aA^
Millet grain 2	247.53 ± 0.08 ^bB^	37.67 ± 0.00 ^bcA^	16.27 ± 0.00 ^bA^	2.33 ± 0.01 ^aA^	85.53 ± 0.02 ^cA^	688.25 ± 0.10 ^aA^
Millet grain 3	299.53 ± 0.32 ^abB^	36.67 ± 0.00 ^cA^	15.40 ± 0.00 ^bcA^	2.13 ± 0.01 ^aA^	83.47 ± 0.02 ^cA^	694.32 ± 0.03 ^aA^
Millet grain 4	310.40 ± 0.32 ^abB^	38.67 ± 0.00 ^abA^	15.27 ± 0.00 ^cA^	2.20 ± 0.01 ^aA^	99.13 ± 0.05 ^bA^	696.39 ± 0.06 ^aA^
Millet grain 5	359.87 ± 0.43 ^aA^	39.07 ± 0.01 ^aA^	17.33 ± 0.00 ^aA^	2.53 ± 0.00 ^aA^	109.47 ± 0.04 ^aA^	697.09 ± 0.40 ^aA^

Note: Data were shown as the mean ± SD values (*n* = 3, mg/kg dry basis). Significant differences between table entries are explained in the footnote of Table 2.

## Data Availability

The data presented in this study are available on request from the corresponding author.
